# *Streptomyces coelicolor*-plant association facilitates ergothioneine uptake in *Triticum aestivum*

**DOI:** 10.3389/fmicb.2025.1637050

**Published:** 2025-07-18

**Authors:** Alexandra Pipinos, Jinjun Kan, Andrew Smith, Gladis Zinati, Harsh Bais

**Affiliations:** ^1^Department of Plant and Soil Sciences, University of Delaware, Newark, DE, United States; ^2^AP Biopharma, University of Delaware, Newark, DE, United States; ^3^Stroud Water Research Center, Avondale, PA, United States; ^4^Rodale Institute, Kutztown, PA, United States

**Keywords:** endophyte, ergothioneine, rhizosphere, roots, wheat

## Abstract

The growing market of agricultural biologicals as alternatives to synthetic crop chemicals is driven by their ability to improve soil health, reduce carbon footprints, enhance crop yield and quality, and help counter declining protein levels in cereal crops linked to climate change and soil degradation. Ergothioneine (EGT), an amino acid with recognized nutraceutical and micronutrient properties, has gained popularity for its anti-inflammatory and antimicrobial properties on human health. While plants and humans cannot biosynthesize EGT, its production by *Streptomyces coelicolor* presents as a promising bio-stimulant to support overall plant and human health. Our study investigates the potential for *S. coelicolor* M145 to enhance EGT levels in spring wheat (*Triticum aestivum*). Results confirmed successful EGT extraction from bacterial cell extracts and plant tissues. The bacterial cells grown in nutrient rich media showed significant levels of EGT post day 7 of incubation, with an average of 0.32 μM of EGT, while bacteria growing in the limiting nutrient condition produced an average of 0.27 μM EGT. In parallel, wheat plants inoculated with *S. coelicolor* and extracted for EGT on day 10 post incubation, showed higher shoot EGT content (0.1168 ± 0.071 μM) in bacteria treated plants. Additionally, a fluorescent confocal microscopy staining and imaging protocol showed bacterial colonization on *T. aestivum* and its potential as a root endophyte. Following root inoculation, *S. coelicolor* was observed to inhabit roots, shoots, and internodes of *T. aestivum*, suggesting its potential endophytic lifestyle on host plants. Our data showed that *S. coelicolor*-associated wheat plants produce EGT *in planta*. Overall, our findings establish a direct link between soil and human health through rhizosphere colonization by *S. coelicolor* and *in planta* production of EGT, suggesting an alternate route to enhance protein concentration in crop plants.

## 1 Introduction

The connection between plant nutrition and human health is a critical area of research, especially as the global population is projected to exceed 10 billion by 2050 (El-Ramady et al., 2022). To address the growing demand for nutrient-rich food, researchers are focusing on fortifying the world’s most widely cultivated crops—such as wheat, soybean, corn, and rice—with essential nutrients and bioactive compounds (Huang et al., 2020). The role of microbial inoculum as biostimulant and biofertilizers is growing and gaining more attention for improving both plant and soil health.

Compared to synthetic fertilizers, agricultural biologics, introduced in the mid-20th century, are natural substances or biological organisms that enhance overall plant health and growth (Priya et al., 2023). With a projected growth to reach around $24.6 billion by 2027, agricultural biologics or microbial inoculum synthetic communities (Syncom) are emerging as a promising alternative to synthetic treatments, while synthetics have come under scrutiny due to their environmental and health impacts (Shayanthan et al., 2022). Agriculture accounts for 12% of global greenhouse gas (GHG) emissions, with a significant portion attributed to dinitrogen monoxide released from nitrogen compounds in chemical fertilizers (Basheer et al., 2024). Moreover, synthetic inputs in crop production may pose risks to human health, having associations with an increased likelihood of certain disease development, including increased risk of asthma, chronic obstructive pulmonary disease (COPD), and lung cancer (Dhankhar and Kumar, 2023; Basheer et al., 2024). In fact, the European Union (EU) has introduced policy measures through the Farm to Fork strategy, established in 2020. This strategy aims to achieve targets such as a 50% reduction in pesticide use and risk, in addition to 20% reduction in chemical fertilizer use by 2030 (Silva et al., 2022). With chemical-based inputs becoming less frequently used, agricultural biologics offer a more sustainable approach to enhancing crop nutrition while minimizing harmful effects on the environment and human health (Priya et al., 2023). Agricultural biologics are often categorized into three main subgroups: biofertilizers, biopesticides, and biostimulants (Miranda et al., 2024).

Plant biostimulants are substances or microorganisms that can be added to plants to increase nutrient efficiency, abiotic stress tolerance, crop quality traits, and other beneficial impacts to overall crop health (du Jardin, 2015). In addition, biostimulants such as humic acids and seaweed extracts have demonstrated the potential to increase plant growth (Ampong et al., 2022; Ali et al., 2021). In addition to seaweed extract, which remains the most extensively studied component of biostimulants, other substances that can function as biostimulants include amino acids, microbials, plant extracts, and organic acids (Ali et al., 2021). Key microbial biostimulants include Plant-Growth Promoting Rhizobacteria (PGPRs), which, in many cases, positively impact plant health with or without directly providing nutrients (du Jardin, 2015).

Actinobacteria represent a varied phylum of Gram-positive, filamentous bacteria that reside in soil and play a crucial role in nitrogen fixation, phosphate solubilization, iron acquisition, and the overall development of crops (Sathya and Ushadevi, 2014). Among this group, *Streptomyces* stands out for its remarkable antimicrobial characteristics and its ability to produce bioactive compounds that promote plant growth (Viaene et al., 2016; Nazari et al., 2023). As natural soil dwellers, *Streptomyces* colonize the rhizosphere both externally and endophytically, improving soil fertility and nutrient uptake (Ge et al., 2024; Vurukonda et al., 2020). Interest in these microbes has grown as declining crude protein levels in cereal crops—driven by climate change, plant breeding, declines in soil organic matter, and changes in environmental legislation—have raised concerns about malnutrition and nutrient deficiencies in vulnerable populations (Styczen et al., 2020). This decline in crude protein content in agricultural crops has further contributed to global nutritional challenges (Medek et al., 2017; Seltenrich, 2017; Styczen et al., 2020). Therefore, exploring methods to enhance protein levels in common cereal crops has become a critical focus in agricultural research.

Ergothioneine (EGT) is a unique, naturally occurring amino acid with potent antioxidant and anti-inflammatory properties that are beneficial to human health (Borodina et al., 2020). Examples of EGT’s potential linkages in health benefits include its association with reduced risk of cardiovascular disease and mortality (Smith et al., 2020). Additionally, increased EGT consumption has been linked to healthy cognitive aging (Tian et al., 2023). EGT also exhibits protective qualities for the skin, including improving skin hydration, enhancing elasticity, and reducing trans-epidermal water loss (Zhang, 2023). These benefits highlight its increasing popularity in skincare research. Although EGT offers significant benefits to human health, it is neither produced in humans nor plants (Carrara et al., 2023). As a result, EGT must be obtained through diet, either from food sources or in supplement form. While dietary sources of EGT are primarily limited to mushrooms, a few fungi and bacteria have adapted the ability to biosynthesize it. Notably, Actinomycetes, Cyanobacteria, and Methylobacteria are the only known bacteria groups capable of biosynthesizing EGT (Borodina et al., 2020). Some fungal species such as *Neurospora crassa*, *Cordyceps militaris*, and mushroom fruiting bodies also produce EGT (Chen et al., 2022). A member of the Actinobacteria class, *Streptomyces coelicolor* M145 contains the five-gene biosynthetic cluster (*egtA*, *egtB*, *egtC*, *egtD*, and *egtE*) to biosynthesize EGT (Nakajima et al., 2015). Therefore, considering *S. coelicolor*’s role as a PGPR and its potential ability to produce EGT, using the organism as a biostimulant to enhance EGT levels in a popular cereal crop-compensating for protein loss- is of high interest.

To advance our understanding of EGT production in *S. coelicolor* M145 and its potential as a biostimulant for enhancing crop health and nutrition, we conducted controlled experiments to evaluate *S. coelicolor* both independently and in conjunction with the inoculation of a widely consumed cereal crop, spring wheat (*Triticum aestivum*). We speculated that associating plants with a EGT producing microbe such as *S. coelicolor* may facilitate EGT uptake in plants, thereby leading to enhanced nutrient content in plants. Our data showed that the presence of EGT in plants post-inoculated with *S. coelicolor*. We demonstrated that *S. coelicolor* functions as a biostimulant, enhancing EGT concentration in *T. aestivum* plants. Our findings support the use of crop biologicals as a viable strategy to increase overall protein concentration, addressing the growing concern of protein deficiency in staple crops.

## 2 Materials and methods

### 2.1 Chemicals

L-Ergothioneine (L-EGT, purity > 99.99%) was obtained from MedChemExpress (Monmouth Junction, NJ). Ammonium acetate, methanol (For HPLC, ≥ 99.9%), water (HPLC grade), and acetonitrile were purchased from Sigma-Aldrich Inc. (St. Louis, MO). For microscopy staining, Wheat Germ Agglutinin (WGA) 594 was obtained from Thermo Fisher Scientific (Waltham, MA) and the calcofluor white stain was obtained from Sigma-Aldrich Inc. (St. Louis, MO).

### 2.2 Instruments

The Xevo-G2-S QTof system by Waters™ (Milford, Massachusetts) was utilized for Ultra-Performance Liquid Chromatography (UPLC) of EGT sample analysis. The Andor Dragonfly confocal super resolution microscope by Oxford Instruments (Abingdon, United Kingdom) was used in the fluorescent confocal microscopy analysis.

### 2.3 Plant material

*Triticum aestivum* variety Surpass Spring seeds were obtained from South Dakota State University (Brookings, SD), and were stored long-term in the dark at room temperature inside their original cloth bag. The seeds were sanitized prior to plating, and the husks were removed by light force. Using sterilized forceps, wheat seeds were transferred to petri plates containing 25 mL of full-strength Murashige and Skoog (MS) (Murashige and Skoog, 1962) media per plate. The plates were sealed with micropore tape or parafilm and incubated under a 12-h photoperiod and 25% humidity at 26°C for 7 days to germinate. A total of 10 plants were analyzed per time point (day 5, 7, and 10) for biomass, root colonization, and EGT analysis. The whole experiment was repeated twice with 10 biological replicates.

### 2.4 Bacterial culture

*Streptomyces coelicolor* M145 strain was donated by Matthew Traxler lab at the University of California, Berkeley. Using a sterile inoculation loop, the culture was streaked onto a solid, soy-flour mannitol (SFM) medium plate. The *S. coelicolor* plates were sealed with micropore tape and incubated at 30°C for 3–5 days until gray-white colonies appeared. For longer-term storage, a 20% glycerol stock was prepared from the original plates and stored in a −80°C freezer.

### 2.5 Preparation of standard solution and standard curve

To create a 10 mM stock solution of L-EGT, 2.293 mg of L-EGT was dissolved in 1 mL of 50% methanol extraction solvent. The stock solution was serial diluted using the extraction solvent to prepare standard solutions at a concentration range of 0.01–10 μM. These standards were used to generate a calibration curve. EGT presence was confirmed using MS/MS tandem mass spectrometry to confirm precursor ion along with product ion presence (Supplementary Figure S1). To account for variations in sample matrices, separate standard curves were generated to determine an appropriate limit of quantification (LOQ) for all sample extract experiments.

### 2.6 Intracellular cell-extraction from *Streptomyces coelicolor* M145 and MS/MS tandem mass spectrometry quantification

Bacterial cells were harvested for EGT quantification on days 5, 7, and 10 of incubation. Approximately 10 mg of the bacterial inoculum was removed from the culture plate and transferred to a centrifuge tube containing 10% acetonitrile (1 mL). The sample was vortexed for 10 s, then sonicated for 10–15 min. After sonication, the sample was transferred to a centrifuge (5430/5430R centrifuge, Eppendorf, Hamburg, Germany) at 4°C for 10 min at 12,000 × g. The supernatant was then transferred to a falcon tube. To ensure all EGT was collected, 1,000 μL of 10% acetonitrile was added to the centrifuge tubes and re-centrifuged under the same conditions and the extra supernatant was collected. The samples were lyophilized for 2 days and were then dissolved in 50% methanol. The samples were filtered, and a spiking solution was added to each sample. The samples were then subjected to Ultra-Performance Liquid Chromatography tandem mass spectrometry analysis (UPLC–MS/MS).

### 2.7 *Streptomyces coelicolor* M145 inoculation on surpass spring wheat

For the inoculation and colonization experiment, *T. aestivum* seeds were sterilized and plated on full strength MS medium. The seeds were left to germinate under a 12-h photoperiod at 26°C and 25% humidity for 7 days. After the spring wheat seeds germinated by day 7, the plants were transferred to 10% MS and were inoculated with 10^6^ cells/mL of *S. coelicolor* suspension. Control plants were incubated with a bacteria-free suspension.

### 2.8 Ergothioneine extraction from inoculated surpass spring wheat

The plants were harvested on days 5 and 10 post-inoculation of *S. coelicolor*. For each time point, three biological replicates were measured. Within each biological replicate, three technical replicates were performed, resulting in a total of nine technical replicates per treatment. For the EGT extraction, a root or shoot sample was ground to a powder using liquid nitrogen and transferred to a tube containing 10% acetonitrile. The samples were briefly vortexed, sonicated for 10 min, then centrifuged at 4°C for 10 min at 12,000 × g. Once centrifugation was complete, the supernatant was transferred to a falcon tube. The pellet was re-extracted with more 10% acetonitrile, vortexed, centrifuged under the same conditions, and the supernatant was collected. The samples were then lyophilized for 48–72 h. The lyophilized product was then dissolved in 50% methanol, filtered, and subjected to Ultra-Performance Liquid Chromatography tandem mass spectrometry analysis (UPLC–MS/MS).

### 2.9 EGT detection by UPLC–MS/MS and overall protein concentration assay

EGT detection was carried out on a Waters Xevo G2-S QTof, with an electrospray ionization probe operated in positive mode. A custom protocol was developed in-house for EGT detection and quantification. Compound separation was conducted on an Intrada Amino Acid column, (50 mm x 3.0 mm, standard pressure) with (solvent A)- acetonitrile containing 0.1% formic acid, and (solvent B)-100 mM ammonium acetate (Beelman et al., 2021). The flow rate for solvent A and solvent B were run at a consistent 0.5 mL/min. Solvent A was run at 95% from the initial time until 4 min, then reduced to 5% between 4 and 4.5 min. At 4.5 min, solvent A was increased to 95% until the end of the run, to 6 min. Solvent B was run at 5% from the initial time to 4 min, then adjusted to 95% from 4 to 4.5 min. At 4.5 min, solvent B was reduced to 5% until 6 min. The autosampler was maintained at 4.0°C, and the column temperature was managed at 40°C. EGT analysis was directed using the Waters MassLynx software (version 4.2), where EGT peaks were analyzed and the area under the curves were integrated to determine the concentration of EGT. A Bradford assay using the BioRad Quick Start™ Bradford Protein Assay (Item #: 5000201) was performed to measure protein levels in plant roots and shoots (Kielkopf et al., 2020). Spring wheat plants inoculated with ~10^6^ cells/mL of *S. coelicolor*, along with control plants, were harvested on day 10 post-inoculation. The same protocol for EGT extraction from plants was used for the protein extraction in this assay. The OD_595_ measurements were taken using the Bio-Rad SmartSpec 3000 UV/VIS Spectrophotometer, and the linear regression equation generated from the standard curve was used to calculate the concentration (mg/mL) of each unknown sample.

### 2.10 Fixation, staining, and confocal fluorescence microscopy of plants colonized by *Streptomyces coelicolor*

The plants were harvested on days 5, 7, and 10 post-inoculation of *S. coelicolor*. *Triticum aestivum* plant samples were sectioned into distinct regions [Crown root (root tip region, lateral region, crown region); internodal region; leaf blade] of the plant and transferred to a 24-well plate containing a fixative composed of 25% glutaraldehyde, 4% paraformaldehyde, 100% Triton X-100, and 1x phosphate buffer solution (PBS) (Pasternak et al., 2015). The samples then underwent vacuum filtration for 1 h at 4°C, and the fixative was then removed. Samples were rinsed with phosphate buffer solution (PBS), and 0.2 M glycine was added into each well, where the samples were placed in a fridge (4°C) overnight. Plant samples were again washed three times with PBS and stained using Calcofluor white and Wheat Germ Agglutinin 594 (WGA594). Finally, solution Scale P (6 m urea, 30% (v/v) glycerol, and 0.1% (v/v) Triton X-100 in 1X PBS pH 7.4) was introduced into each well. The samples were stored at room temperature in a dark room until fluorescent confocal microscopy imaging. Z-stacking was performed for each region of the plant sample displaying colonization of bacteria using the Leica DMi8 widefield scope as a base coupled with the Andor Dragonfly 600 Spinning Disc Confocal imaging scope located in the University of Delaware Bioimaging center. Calcofluor white was excited using a 405 nm laser with emission captured at 446 nm, and Alexa fluor 594 was excited at 561 nm with emission captured at 594 nm. Z stacks were analyzed using Imaris Microscopy Image Analysis Software by Oxford Instruments.

### 2.11 Endophyte assay

Treated *Triticum aestivum* shoots and internodes along with untreated control plants were sectioned into various regions using a sterile scalpel and forceps per the published protocol (Worsley et al., 2020). The cut plant samples were briefly immersed in 50% ethanol for 15 s, rinsed with sterile water, and placed onto soy-flour mannitol (SFM) plate. Samples were incubated at 30°C for 3–5 days and subsequently examined for *S. coelicolor* growth.

### 2.12 Statistical analysis

The data was analyzed using GraphPad Prism (GraphPad Prism, version 10.4.0, Boston, MA). The following tests were performed for data analysis: One-way ANOVA statistical test paired with the Brown-Forsythe and Welch test, Dunnett’s T3 multiple comparisons test, Welch’s test, all with a significance level of *p* < 0.05.

## 3 Results

### 3.1 Inoculation of *Streptomyces coelicolor* M145 on *Triticum aestivum* enhances overall root and shoot fresh weight

**Figure 1:**
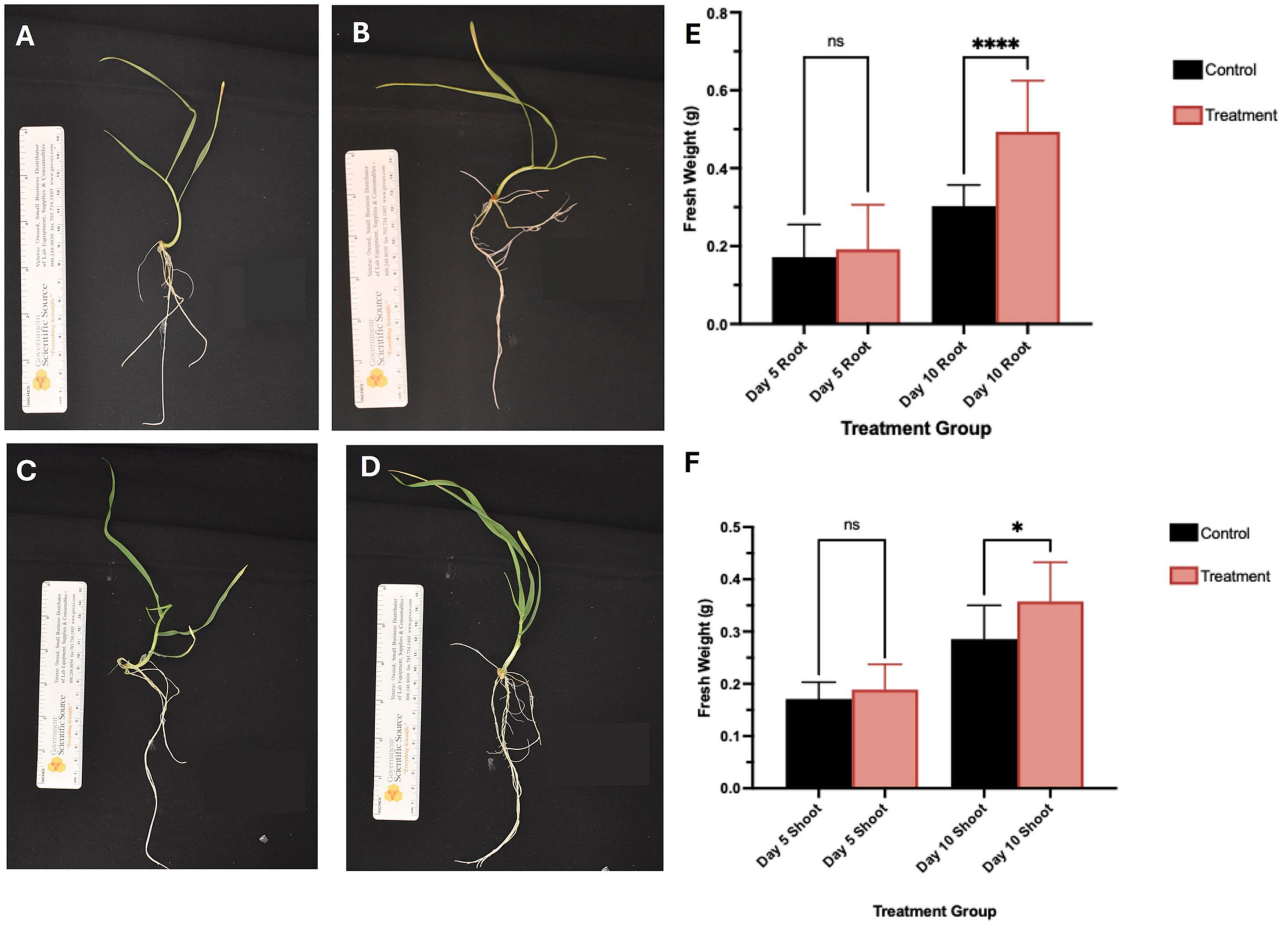
Representative images of plants at day 5 and 10 post-inoculation on the day of harvest, the panels **(A,B)** showed control and *S. coelicolor* inoculated plants on day 5. The panels **(C,D)** show control and *S. coelicolor* inoculated plants on day 10. Scale bar = 1.0 cm. **(E,F)** Shoot fresh weight (g) of *Triticum aestivum* plants in control and treatment groups post-inoculation with *S. coelicolor* M145. The panels **(E,F)** show morphological traits in control and *S. coelicolor* inoculated plants on day 5 and 10. Asterisk indicates statistically significant differences (*p* < 0.05) between treatments and the untreated control.

Representative images of the harvested plants are shown in Figures 1A–D significant increase in root and shoot biomass was observed 10 days post-inoculation with *S. coelicolor* M145 (see Figures 1E,F; *p* < 0.05). These results suggest that *S. coelicolor* acts as a plant growth-promoting rhizobacterium (PGPR) by enhancing *T. aestivum* fresh weight in both roots and shoots.

### 3.2 Exogenous EGT supplementation induces morphological changes in *Streptomyces coelicolor* M145

**Figure 2:**
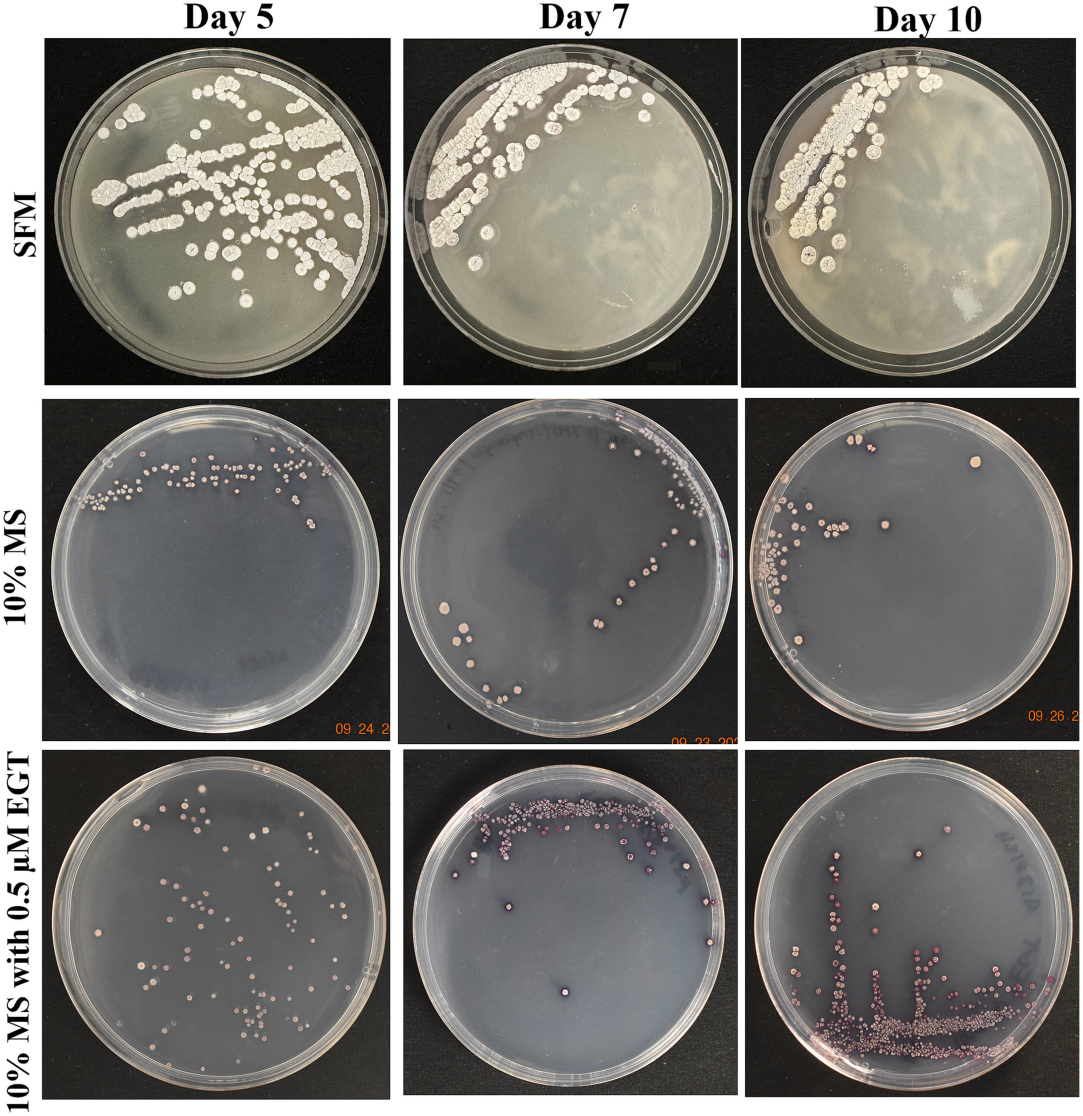
Growth of *Streptomyces coelicolor* on day 5, 7, and 10 across three media types. *Streptomyces coelicolor* was plated on soy-flour mannitol (SFM), 10% Murashige and Skoog (MS), and 10% MS supplemented with 0.5 μM L-EGT. The images illustrate morphological changes and growth patterns under varying nutritional conditions.

Growth of *Streptomyces coelicolor* M145 varied across varying media conditions, with morphological characteristics including pigmentation being documented (Figure 2). When *S. coelicolor* was grown on its preferred rich media, SFM, morphological characteristics of the cells were relatively consistent across days 5, 7, and 10 of growth. The colonies were relatively larger, with clear and consistent mycelial formations visible in each colony. The colonies appear gray/white throughout all three time points, with one noticeable feature on day 10 where one-two colonies are shown to be producing actinorhodin (purple color), a secondary metabolite that is oftentimes produced when *S. coelicolor* is under nutrient-limiting or stressful conditions. When *S. coelicolor* was grown under limiting condition with 10% MS, the size of the colonies averages a smaller diameter compared to the SFM grown cells, with inconsistency in diameter (see Figure 2). The recorded colonies were still gray/white in color, but there was a lack of clear mycelial growth compared to the SFM cells (see Figure 2). The production of actinorhodin was seen to progress with inoculation time under limiting growth conditions (Figure 2). To evaluate the role of exogenous EGT, *S. coelicolor* cells grown on 10% MS media were supplemented with 0.5 μM L-EGT. On day 5, for *S. coelicolor* cells have signs of actinorhodin production both intracellular and extracellularly (see Figure 2). Similarly to the 10% MS cells, there is inconsistent diameter size of the bacterial colonies in the 10% MS with L-EGT. Some of the colonies appear gray/white. By day 7 of growth, the cells appear to have produced significantly higher levels of actinorhodin compared to day 5. The day 10 image appears similarly to day 7, with most of the colony’s purple from actinorhodin synthesis, relatively small colony diameters compared to SFM and 10% MS cells, and a lack of clear morphological development such as mycelial formations. Overall, it can be interpreted that providing exogenous 0.5 μM L-EGT to a minimal media induces elevated stress to *S. coelicolor* cells by the visually enhanced production of actinorhodin.

### 3.3 EGT production in *Streptomyces coelicolor* M145 grown under different media conditions

**Figure 3:**
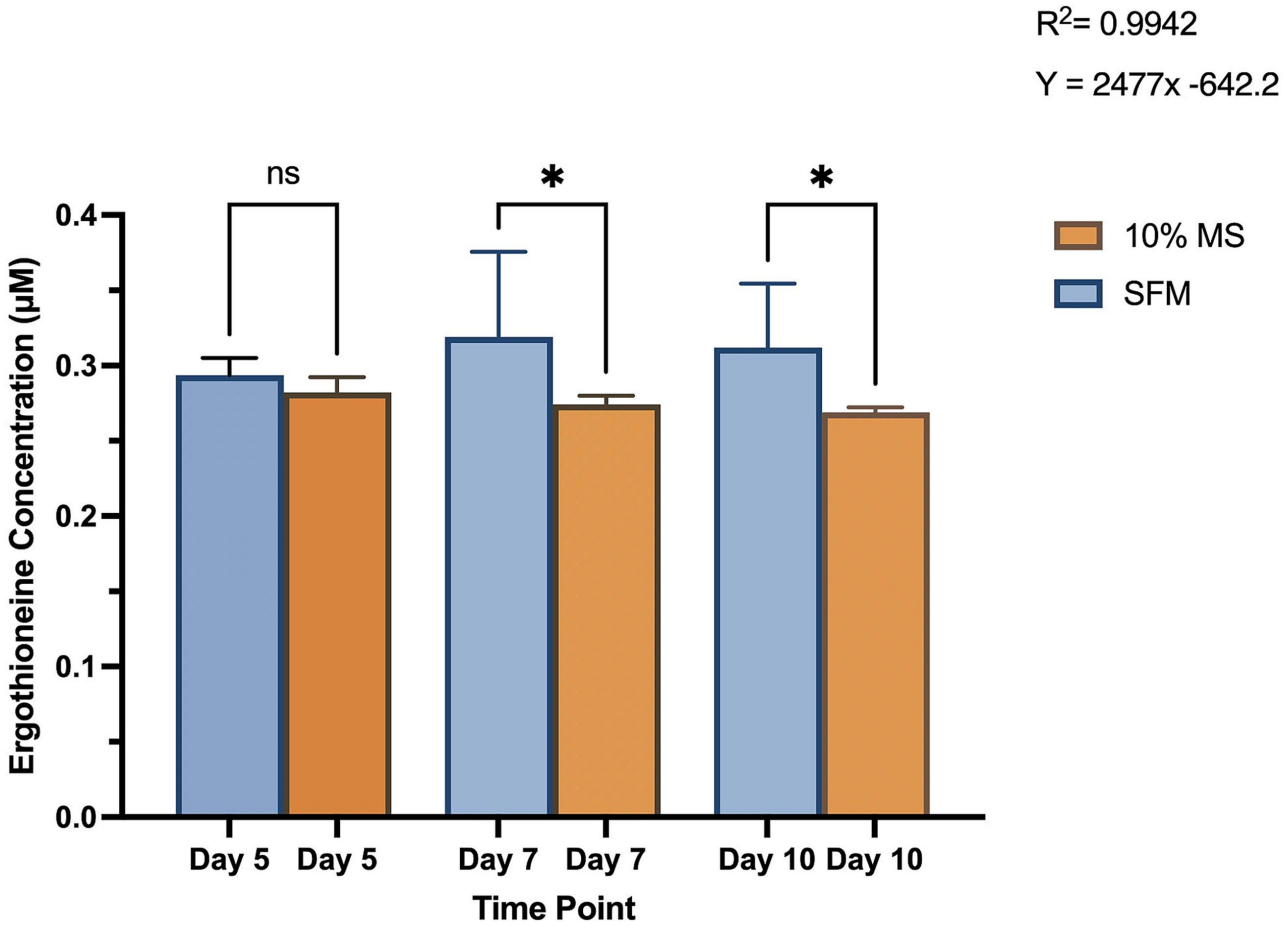
Intracellular ergothioneine (EGT) levels in *Streptomyces coelicolor* M145 cells grown on a plant growth medium, 10% Murashige and Skoog (MS) and Soy-Flour Mannitol (SFM) on days 5, 7, and 10 of growth. EGT levels are displayed in μM. Asterisks indicate statistically significant differences (*p* < 0.05) between treatments.

Growth of *S. coelicolor* varied across different media conditions. On day 5 of harvest, SFM and 10% MS cells produced very similar levels of EGT, where cells grown on 10% MS averaged insignificantly lower concentrations of EGT compared to SFM (see Figure 3). By day 7, there was a statistically significant difference in EGT production comparing the two media conditions. *S. coelicolor* cells grown on SFM produced an average of ~0.32 μM of EGT, while 10% MS cells were producing an average of 0.27 μM (see Figure 3). The same level of statistical significance is shown comparing day 10 under both the media conditions (SFM and 10% MS), where *S. coelicolor* cells grown on SFM revealed higher EGT production compared to 10% MS (average ~0.31 μM SFM, ~ 0.27 μM 10% MS) (SFM Day 5: 0.2937 ± 0.0114 μM; SFM Day 7: 0.3191 ± 0.057 μM; SFM Day 10: 0.3121 ± 0.0424 μM; 10% MS Day 5: 0.2822 ± 0.010 μM; 10% MS Day 7: 0.2743 ± 0.006 μM; 10% MS Day 10: 0.2690 ± 0.003 μM; *p* = 0.0050). The data suggests that EGT production in *S. coelicolor* is modulated with the culture period and media conditions. The data also reveals that a rich media condition may facilitate more EGT production in *S. coelicolor* cells.

### 3.4 Bioimaging reveals different lifestyles of *Streptomyces coelicolor* M145 on plant host *Triticum aestivum*

**Figure 4:**
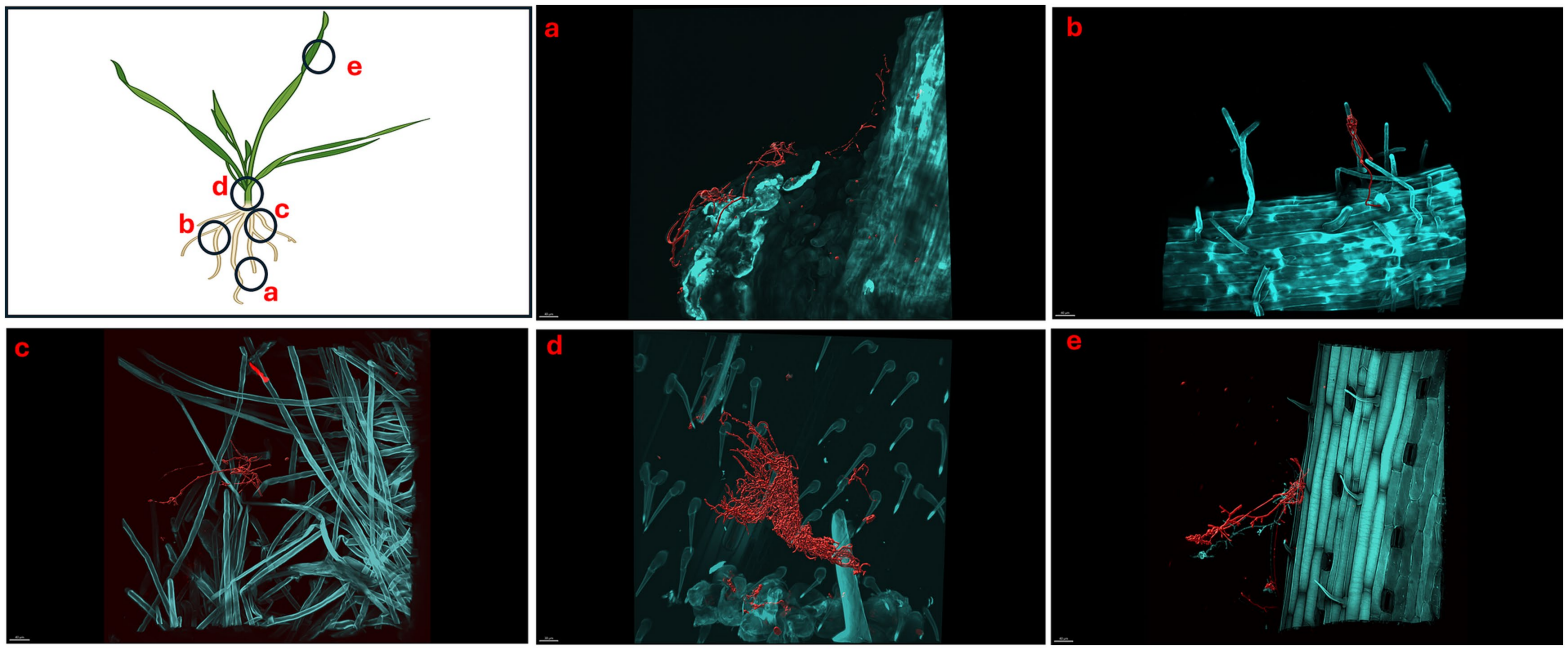
Micrographs showing *Triticum aestivum* plants inoculated with *Streptomyces coelicolor* M145 5-days post treatment. Samples were stained with both calcofluor white and wheat-germ agglutinin (WGA) 594. *Streptomyces coelicolor* M145 is visualized in red. The letters on the schematic (left) shows the sampling regions for microscopy. **(a)** Scale bar = 30 μM, **(b)** scale bar = 30 μM, **(c)** scale bar = 10 μM, **(d)** scale bar = 40 μM, **(e)** scale bar = 30 μM.

**Figure 5:**
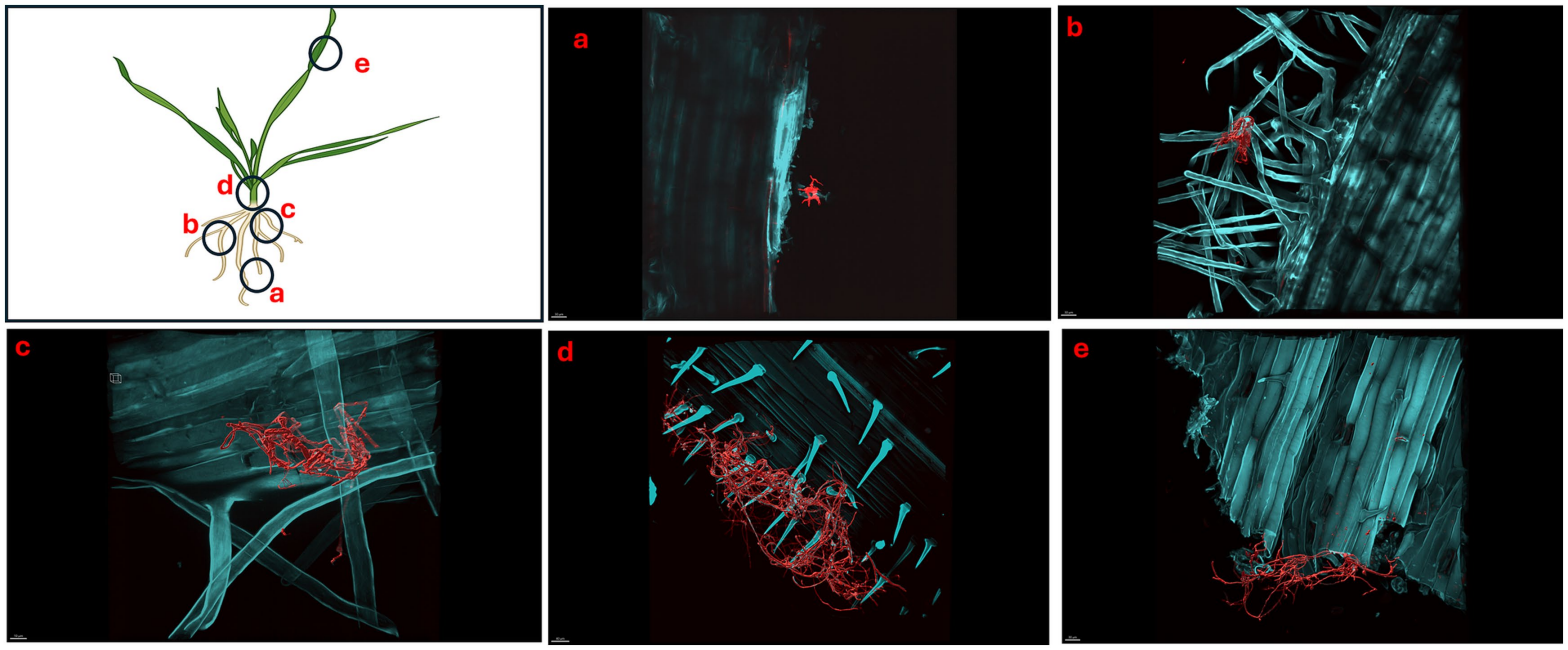
Micrographs showing *Triticum aestivum* plants inoculated with *Streptomyces coelicolor* M145 7-days post treatment. Samples were stained with both calcofluor white and wheat-germ agglutinin (WGA) 594. *Streptomyces coelicolor* M145 is visualized in red. The letters on the schematic (left) shows the sampling regions for microscopy. **(a)** Scale bar = 30 μM, **(b)** scale bar = 30 μM, **(c)** scale bar = 40 μM, **(d)** scale bar = 40 μM, **(e)** scale bar = 30 μM.

**Figure 6:**
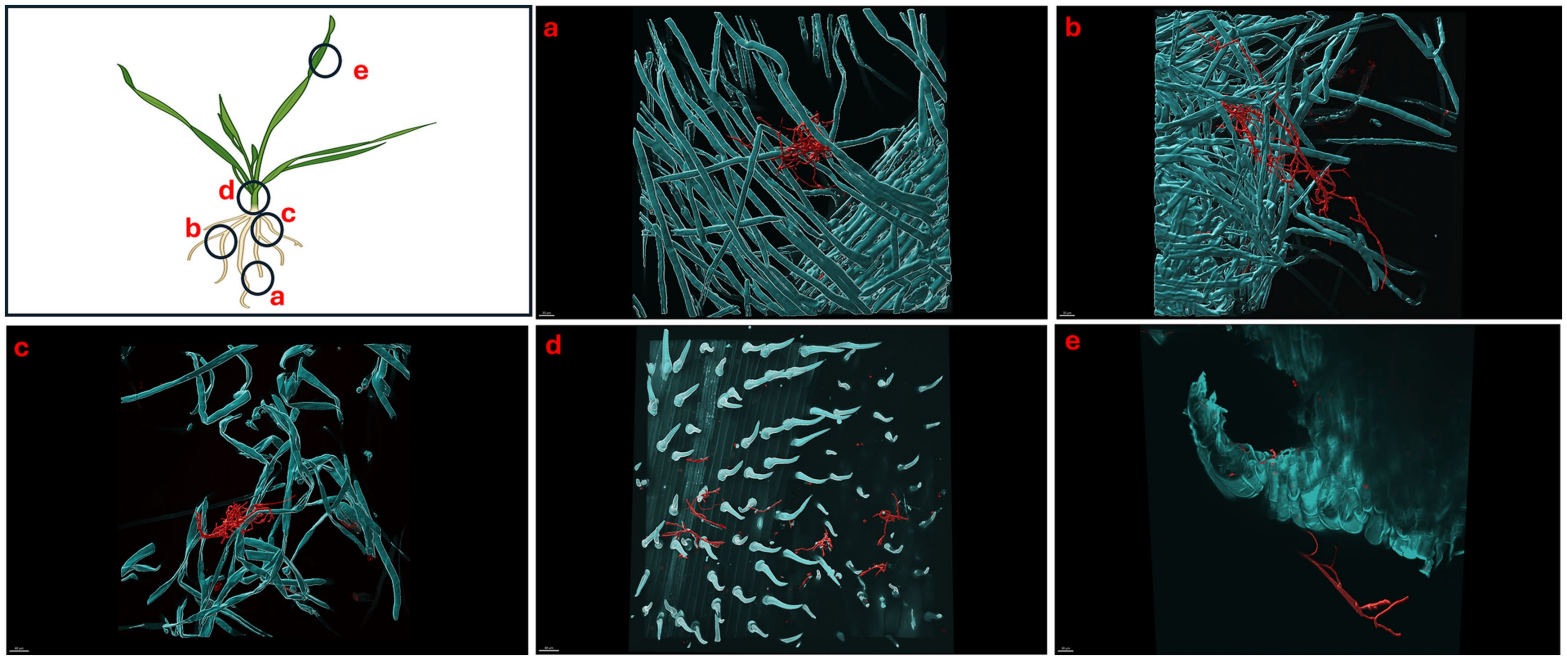
Micrographs showing *Triticum aestivum* plants inoculated with *Streptomyces coelicolor* M145 10-days post treatment. Samples were stained with both calcofluor white and wheat-germ agglutinin (WGA) 594. *Streptomyces coelicolor* M145 is visualized in red. The letters on the schematic (left) shows the sampling regions for microscopy. **(a)** Scale bar = 40 μM, **(b)** scale bar = 40 μM, **(c)** scale bar = 40 μM, **(d)** scale bar = 30 μM, **(e)** scale bar = 40 μM.

We showed that *S. coelicolor* biosynthesizes EGT and inoculation of *S. coelicolor* on host plants (wheat) promotes growth, next we evaluated the colonization patterns of *S. coelicolor* on wheat plants using live imaging. *Triticum aestivum* plants inoculated with ~10^6^ cells/mL of *S. coelicolor* were harvested for confocal fluorescence microscopy on different days post inoculation (Figures 4–6). The control untreated plants were imaged to reveal the gnotobiotic nature of the experimental condition devoid of any contaminants (see Supplementary Figure S2). To monitor the temporal colonization pattern of *S. coelicolor* on wheat plants, we investigated the different areas of colonization throughout the wheat plants. The regions of interests for colonization are depicted as schematic in Figure 4. We also developed a staining protocol using a fluorescent dye WGA594 for *S. coelicolor*. The regions of interest were divided into five different plant compartments [root tip (a), lateral root (b), crown (c), node (d), and leaf blade (e)] to evaluate the presence of *S. coelicolor* on wheat plants. Plants were imaged days 5, 7, and 10 post inoculation with *S. coelicolor* and different regions were compared for the presence of *S. coelicolor*. As early as day 5 post inoculation, *S. coelicolor* was observed in the root tip and elongation zone, with threads of vegetative hyphae clearly visible and appear to be linking on to a root hair (Figure 4a). Temporally, *S. coelicolor* was observed to be present and associated with the lateral root regions mainly colonizing the root hairs post day 5 of inoculation. Like the root tip region, there are evident vegetative and aerial hyphae formations. However, in this region, the structural formations of *S. coelicolor* are more extensive as compared to the root tip regions (Figure 4b), where a larger colony is seen colonizing the root hairs. The crown region shows more thorough distribution of *S. coelicolor* with visible vegetative mycelia clasping the mature root hairs (see Figure 4c). The node region post day 5 of inoculation showed presence of *S. coelicolor* colonizing the trichomes (see Figure 4d). Interestingly, when the leaf blades were analyzed post fixing and staining, colonization by *S. coelicolor* was observed in the cut regions (see Figure 4e).

Like day 5, imaging of day 7 and day 10 plants showed the similar pattern of colonization by *S. coelicolor* on wheat plants (see Figures 5, 6). Visible colonization of *S. coelicolor* was observed across all five imaged regions. Beginning at the root tip region, the morphology of *S. coelicolor* appeared more advanced compared to day 5, revealing defined cross-wall formations extending from the vegetative hyphae with clear attachment to root hairs (see Figures 5a, 6). Further, the lateral root formation region (region 5b) imaged on day 7 exhibited extensive growth and advanced morphology of *S. coelicolor* with hyphae formation, demonstrating *S. coelicolor*’s strong attachment to the multiple areas of the root hairs. Further up the root in the crown region revealed a cluster of *S. coelicolor* like those observed in other regions of the root on day 7 post-inoculation. This image, taken further away from the root itself, highlights *S. coelicolor*’s capability to colonize root hairs beyond the immediate vicinity of the root (see Figure 5c). Beyond the root region, the internode was examined for colonization. However, excessive pressure applied to the larger diameter of the internode impaired the full view of structure and morphology of *S. coelicolor*, making the analysis less interpretable. Despite this, the imaging revealed clear cross-wall formations, vegetative hyphae extensions, and evident colonization of *S. coelicolor* around the trichomes of the internodes (see Figure 5d). Finally, the shoot from day 7 post-inoculation was analyzed and revealed less abundant *S. coelicolor* colonizing the *T. aestivum*, positioned at a distance from the shoot (see Figure 5e). Lastly, day 10 *T. aestivum* plants were harvested and imaged (Figure 6). At the root tip region, a substantial colony of *S. coelicolor* was identified, with the bacteria visibly wrapping around the entire left side of the root tip, suggesting that *S. coelicolor* can colonize the root tip itself (Figure 6a). Moving beyond the root tip, the middle root image (region b) showed *S. coelicolor* extensively wrapping around a single root hair, encompassing it from all angles. Within this single *S. coelicolor* colony, two sporulating regions were evident (see Figure 6b). Continuing from the middle root, filamentous *S. coelicolor* were observed intertwining through the root hairs. Although this region corresponds to the mature root area, there appear to be fewer distinct cross-wall formations (Figure 6c). Further beyond the root region, the internode exhibited the most extensive hyphae formations observed in the day 10 analysis. This region displays dense hyphae formations surrounding the trichomes of the internode. Notably, the hyphae curve around the base of the trichomes in multiple areas, which are potential entry points into the wheat plant for ingression (Figure 6d). If *S. coelicolor* exhibits endophytic behavior in this *in vitro* analysis, it is possible that the bacteria are gravitating toward these openings as a mechanism for plant entry. To verify our hypothesis that *S. coelicolor* may harness wheat plants to adapt to endophytic style, we conducted experiments wherein wheat plants were inoculated with *S. coelicolor* at the root level. Leaf blades were harvested post days 5, 7, and 10 days of *S. coelicolor* inoculation. The cut leaf blades were surface sterilized with 50% ethanol for 10 s and placed on rich media. Our data showed that the leaf blades post surface sterilization led to formation and establishment of *S. coelicolor* colonies once plated (Supplementary Figure S3). This data shows two distinct explanations, one that *S. coelicolor* moves extensively on the plant surface to reach the aerial parts or *S. coelicolor* may adapt to an endophytic lifestyle on wheat plants.

### 3.5 Extraction of EGT from *Triticum aestivum* roots and shoots post *Streptomyces coelicolor* M145 inoculation

**Figure 7:**
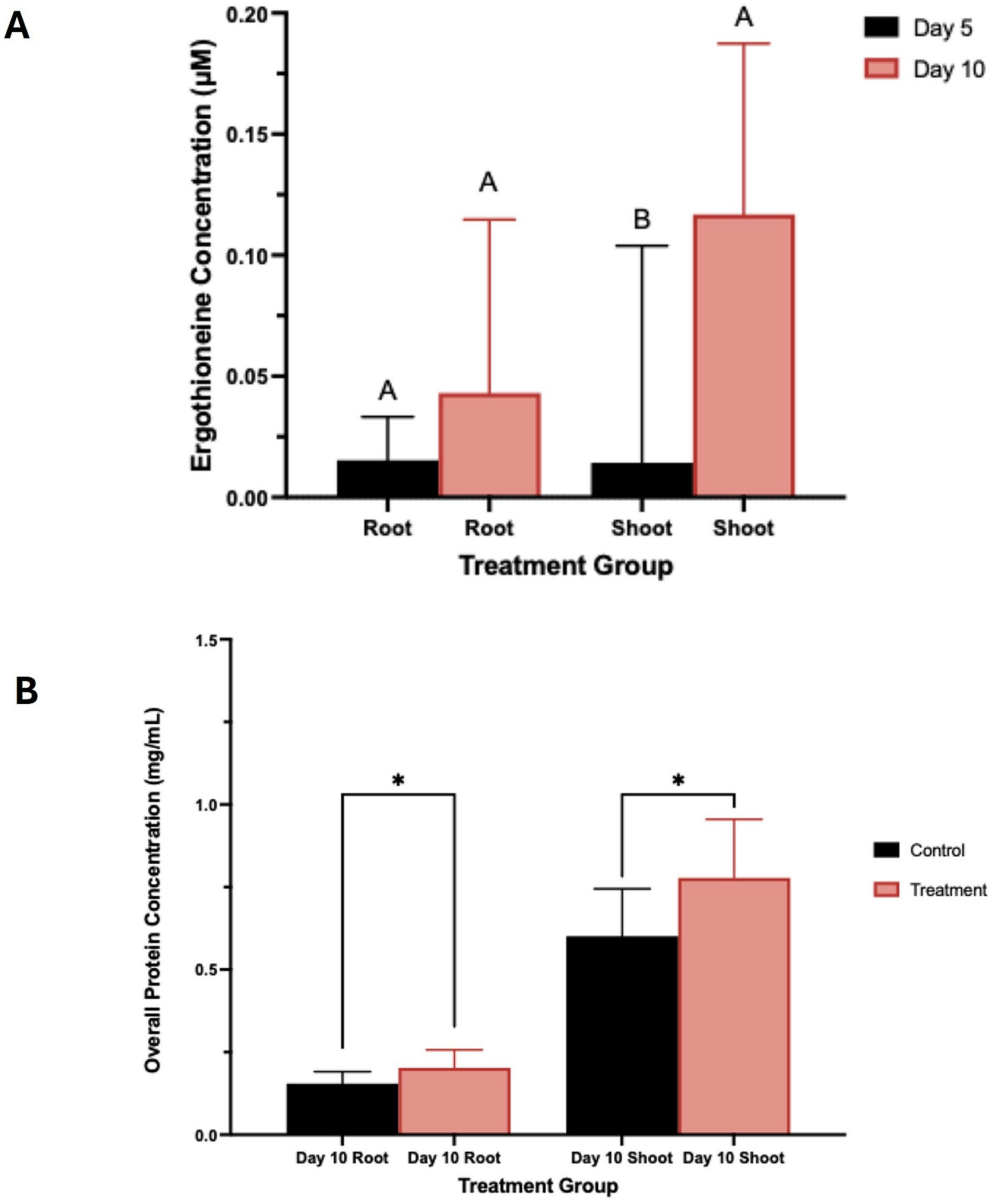
**(A)** Total ergothioneine concentration in wheat plants treated with *S. coelicolor*. Plants were treated with *S. coelicolor* and harvested on day 5 and 10. Roots and shoots were extracted for the total EGT concentration post bacterial treatment. Letters indicate statistically significant differences (*p* < 0.05) between treatments. **(B)** Total protein concentration in wheat plants treated with *S. coelicolor* and harvested on day 5 and 10. Roots and shoots were extracted for the total protein concentration post bacterial treatment. Letters indicate statistically significant differences (*p* < 0.05) between treatments. * indicates significant difference in between the treatments.

*Triticum aestivum* plants inoculated with *S. coelicolor* M145 along with control plants were harvested on days 5 and 10, and EGT was detected and quantified from the roots and shoots of *T. aestivum* (Figures 1, 7A). By day 5, quantifiable amounts of EGT were detectable in both roots and shoots of the plants treated with *S. coelicolor*. On the day 5 roots, trace amounts of EGT were recovered, alongside an increase in EGT on day 10 roots. Statistical analysis revealed the increase was present but not statistically significant (Root EGT content: day 5: 0.0154 ± 0.018 μM; Root EGT content: day 10: 0.0430 ± 0.072 μM; *p* = 0.2538). Meanwhile, the EGT extract from the shoot data showed a slightly different trend, with day 5 shoots containing trace amounts of EGT. By day 10, the shoots had accumulated significantly more EGT, overall indicating that EGT accumulates in a time-dependent manner in both roots and shoots of *T. aestivum* plants inoculated with *S. coelicolor* (Shoot EGT content: day 5: 0.0143 ± 0.090 μM; Shoot EGT content: day 10: 0.1168 ± 0.071 μM; *p* = 0.0184). The untreated control plants extracted for EGT on day 5 and 10 negated presence of EGT (data not shown).

### 3.6 Inoculation of *Streptomyces coelicolor* M145 on *Triticum aestivum* significantly enhances overall protein concentration

In the control *T. aestivum* roots, a lower protein concentration was detected compared to the *S. coelicolor*-treated plants. This result was significant, indicating that treatment of *S. coelicolor* on *T. aestivum in-vitro* leads to an increase in total protein concentration in both aboveground and belowground plant organs (see Figure 7B) (Root Day 10 control: 0.1548 ± 0.036 μM; Root Day 10 treatment: 0.2028 ± 0.054 μM; *p* = 0.0274). Comparatively, the control *T. aestivum* roots/shoots exhibited a lower protein concentration than the *S. coelicolor*-treated plants (Shoot day 10 control: 0.6014 ± 0.143 μM; Shoot day 10 treatment: 0.7775 ± 0.1775 μM; *p* = 0.0400).

## 4 Discussion

### 4.1 EGT biosynthesis declines under nutrient stress conditions

Actinobacteria, cyanobacteria, and some Methylobacteria species are known to biosynthesize EGT (Cumming et al., 2018). Additionally, some fungal species, including *Neurospora crassa*, *Cordyceps militaris*, and mushroom fruiting bodies, also biosynthesize EGT (Chen et al., 2022). A previous study found that EGT levels were quantifiable in both intracellular and extracellular compartments of *Mycobacterium smegmatis* under normal growth conditions, with significantly higher extracellular EGT titers, suggesting active secretion of EGT (Emani et al., 2013). Alternatively, in our study, we focused on quantifying intracellular EGT in *S. coelicolor*, where we hypothesized that growth media conditions play a role in *S. coelicolor* morphology and EGT production. The goal of our experiment was to compare intracellular EGT titers in *S. coelicolor* grown on its optimal medium, to those in a significantly less nutrient-rich medium formulated for plant health. Our study found that, at all three time points of extract harvest, EGT levels were consistently higher when it was grown on a more nutrient rich media. We argue, that EGT being a secondary metabolite, its production is likely favored when primary metabolic needs are sufficiently met. Primary metabolites are essential for cellular growth and development, and typically include amino acids, organic acids, and carbohydrates (Salam et al., 2023). When the primary metabolites are sufficiently produced, the organisms may redirect their resources toward production of secondary metabolites, such as EGT. This indicates that EGT is not involved in the essential survival of *S. coelicolor*, but can present as a stress protector, ultimately providing a competitive ecological advantage over other organisms (Salam et al., 2023). These findings also prove *S. coelicolor*’s metabolic robustness in the production of EGT, making large-scale fermentation processes of this amino acid more effective, resourceful, and potentially more cost productive. The implications of EGT for improving human health, coupled with its role as a secondary metabolite in *S. coelicolor*, suggest that this amino acid could be a promising candidate for utilization in agrochemical and pharmacological applications.

### 4.2 *Streptomyces coelicolor*-plant association and *in planta* EGT production

Previous literature has shown that in soil experiments, low levels of EGT can be detected in plants due to the presence of EGT-producing fungi or bacteria (Tian et al., 2023). Specifically, low levels of EGT have been found in plants such as garlic, wheat, oats, and beans when grown in association with EGT-producing microorganisms (Tian et al., 2023). It is assumed that these microorganisms attach to the plant roots and often function as endophytes, entering the roots and subsequently producing EGT within the plant (Park et al., 2010). Among the Actinobacteria class, a prior study utilized scanning electron microscopy (SEM) to isolate and identify 482 *Streptomyces* strains as endophytes from 28 different plant species. Specifically in spring wheat (*Triticum aestivum*), nine different native *Streptomyces* strains were identified, confirming the idea that *Streptomyces* may act as root endophyte (Ge et al., 2024). Also, z-stacking has been previously implemented in research to study the relationship between motile and non-motile endophytic bacteria and their colonization patterns (Kaduchová et al., 2024). Using this method, the authors observed a high abundance of live bacteria along the periphery of host cells, specifically within the peri-space between the cell wall and plasma membrane (Kaduchová et al., 2024). In our study, we hypothesized that *S. coelicolor* colonization to *T. aestivum* and potential ingression by bacteria may show *in planta* EGT production. As a result, we developed a fluorescent confocal microscopy protocol to analyze *S. coelicolor* colonization and its potential as an endophyte in spring wheat under *in-vitro* conditions. Upon successfully developing a fixation and staining protocol, we determined that *S. coelicolor* utilizes its hyphal extension properties in combination with plant root interactions to distribute itself throughout the three root regions imaged: root tip, middle root, and upper root (maturation zone). In addition to root colonization, we observed that *S. coelicolor* also colonizes the nodal plane and leaf blade regions. Interestingly, in the shoot region, *S. coelicolor* was detected interlacing at the site where the shoot was cut for microscopy preparation. This distribution of *S. coelicolor* to the internodes and shoots can be justified by a variety of reasoning. Since *S. coelicolor* is nonmotile, it typically relies on water, wind, or other organisms for movement (Schlimpert and Elliot, 2023). One possible explanation for the microscopy analysis from the *in-vitro* experiment is that, due to the plant being sealed in the system with a liquid bacterial inoculum, along with consistent transpiration and temperature fluctuations between the petri plate and the external environment, the bacteria may have transferred themselves via water to the shoot regions, moving away from their initial inoculation site at the roots. Alternatively, *S. coelicolor* may have found an entry point and exhibited endophytic behavior, which could explain its presence in the area where the shoot was excised.

Further analysis using LC–MS/MS revealed quantifiable trace levels of EGT in both the roots and shoots of the plant following the inoculation of *S. coelicolor* on *T. aestivum*. By day 10 post-inoculation of bacteria, both the roots and shoots exhibited significantly higher levels of EGT. Similarly, by day 10, fresh weight measurements of both roots and shoots were significantly higher in treated plants compared to controls. These findings confirm our hypothesis that *S. coelicolor* contributes to EGT enhancement of *T. aestivum*. Beyond enhancing EGT levels *in planta*, we aimed to determine whether protein concentration increased in *T. aestivum* inoculated with *S. coelicolor.* A previous study confirmed that inoculation with three different PGPR’s under greenhouse conditions increased overall protein concentration in *Cucumis sativus* L. fruit with respect to the control (Bhardwaj et al., 2024). In our study, *S. coelicolor* inoculation of *T. aestivum in-vitro* significantly enhanced overall protein concentration in both roots and shoots, suggesting that *S. coelicolor* plays a role in enhancing nutrient composition, presumably through its PGPR associated mechanisms including improved nutrient uptake or metabolic regulation.

Conclusion. Taken together, our research demonstrates that the biosynthesis of EGT by *S. coelicolor* suggests its significant role in microbial stress responses, while also highlighting its biotechnological potential. Using the LC-MS/MS method we developed to detect EGT extracted from *S. coelicolor* cells, we found that EGT is biosynthesized as a secondary metabolite at trace levels under both nutrient rich and minimal conditions. Our findings also suggest that *S. coelicolor* inoculation in plants enhance EGT and overall protein concentration, which may be beneficial to fortify cereal plants for enhanced protein concentration in agriculture. Through our development of a confocal fluorescent microscopy protocol, inoculation of *S. coelicolor* on *T. aestivum* resulted in visible colonization of roots, shoots, and internodes. This colonization corresponded with enhanced EGT and biomass levels in both root and shoots. These findings suggest that *S. coelicolor* functions potentially as an endophyte, contributing to EGT biosynthesis and promoting plant growth and nutrient accumulation within the plant. Given the growing relevance of EGT in both agricultural and medical contexts, this research further establishes *S. coelicolor* as a valuable organism for future studies on its potential applications for both plant and human physiological studies. In addition, the role of EGT biosynthesizing soil microbes for plant association opens a new discussion to evaluate the soil to human health continuum in the context of crop fortification by harnessing soil/plant microbiome.

## Data Availability

The original contributions presented in the study are included in the article/Supplementary material, further inquiries can be directed to the corresponding author.
