# Supplementary material for: Streptomyces coelicolor-plant association facilitates ergothioneine uptake in Triticum aestivum
Source: Front Microbiol. 2025 Jul 18;16:1637050. doi: 10.3389/fmicb.2025.1637050 (PMC12313630; doi:10.3389/fmicb.2025.1637050)
Supplement: Supplementary file 1 [file Data_Sheet_1.PDF]

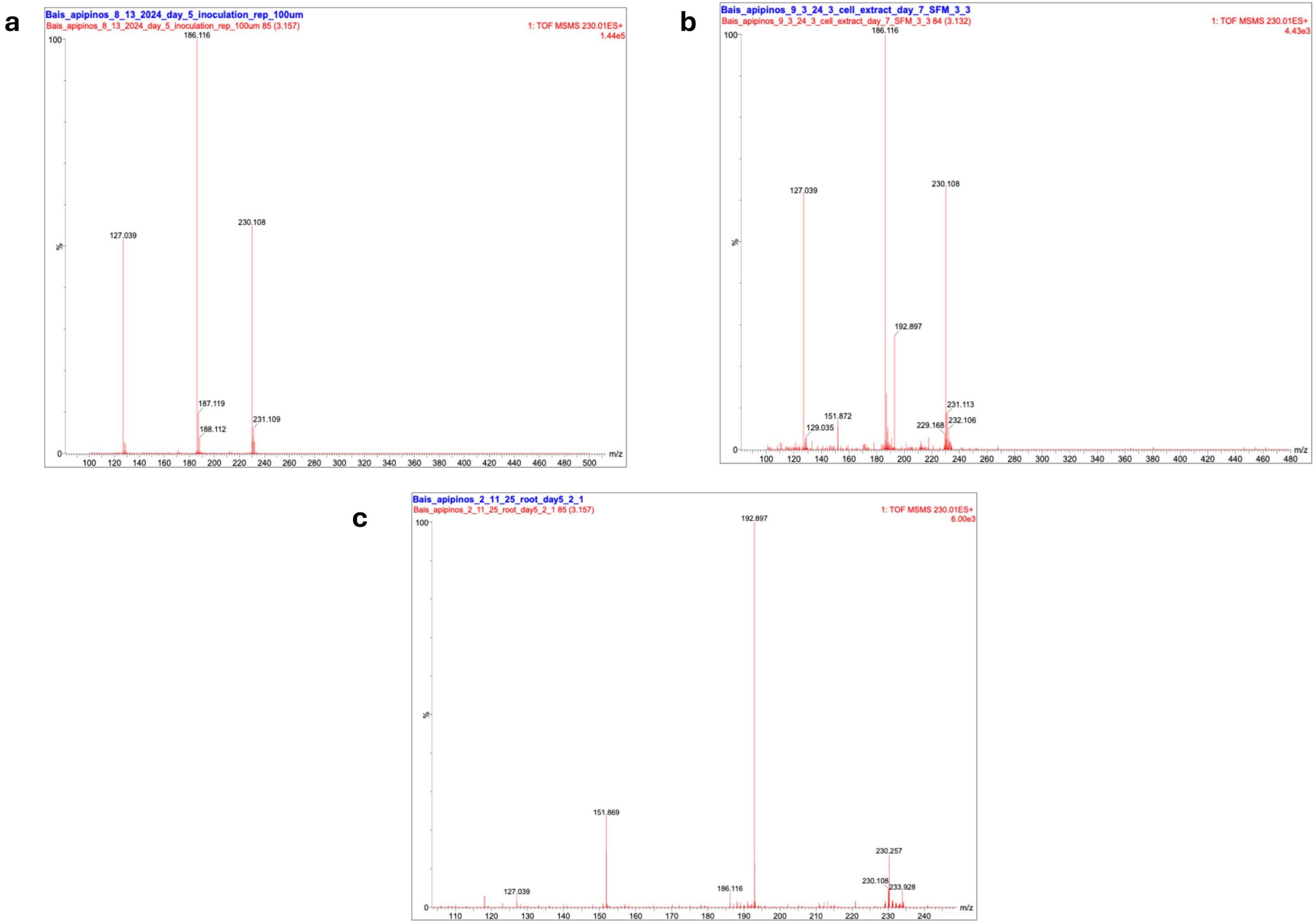

SOM Figure 1 : Resulting mass spectrometry data from the TOF-MS/MS profile of a pure ergothioneine standard (a); *S. coelicolor* cell extracts (b) Intracellular *S. coelicolor* M145 intracellular extracts and (c) wheat root extracts.

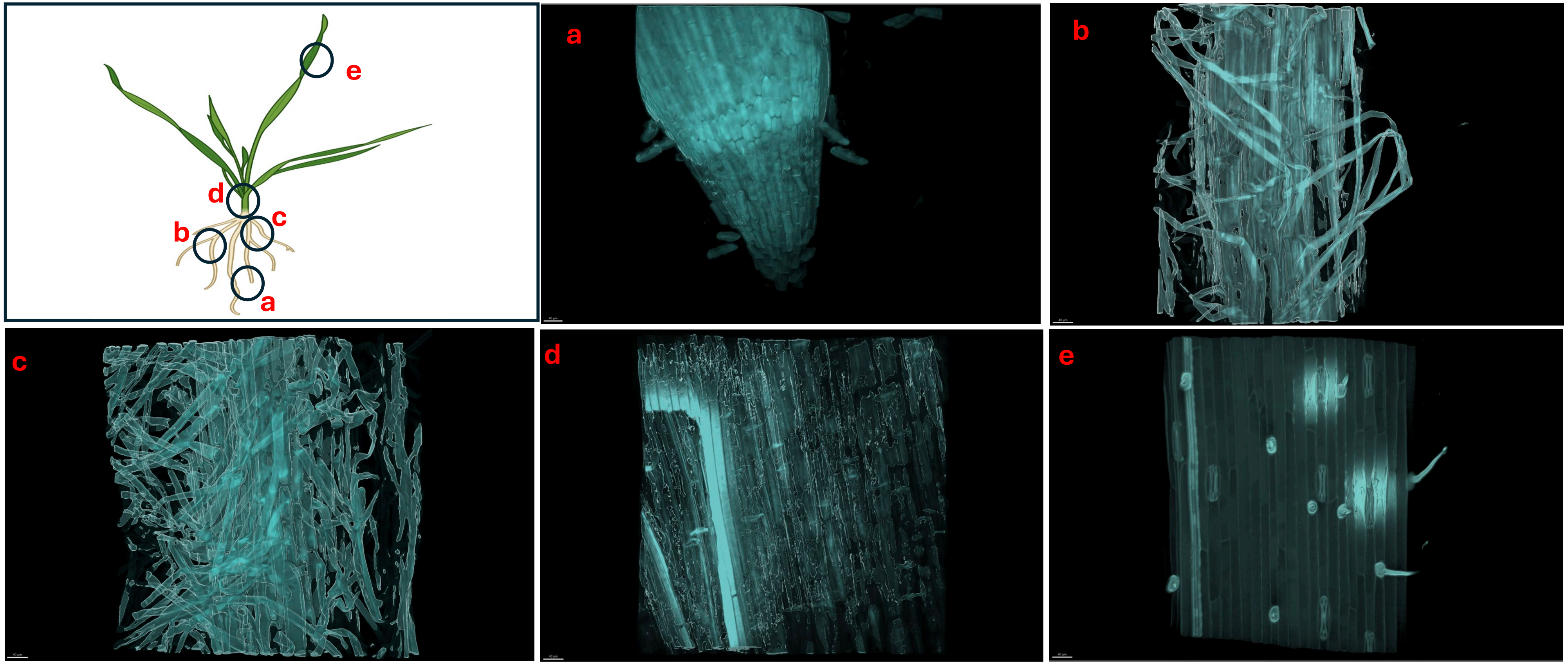

SOM Figure 2: Microscopy of *Triticum aestivum* plants untreated with *Streptomyces coelicolor* M145. The images were taken post 10 days of growth cycle. Samples were stained with both calcofluor white and wheat-germ agglutinin (WGA) 594. The letters on the schematic (left) shows the sampling regions for microscopy. (A) scale bar = 40 µM, (B) scale bar = 40 µM, (C) scale bar = 40 µM, (D) scale bar = 40 µM, (E) scale bar = 40 µM.

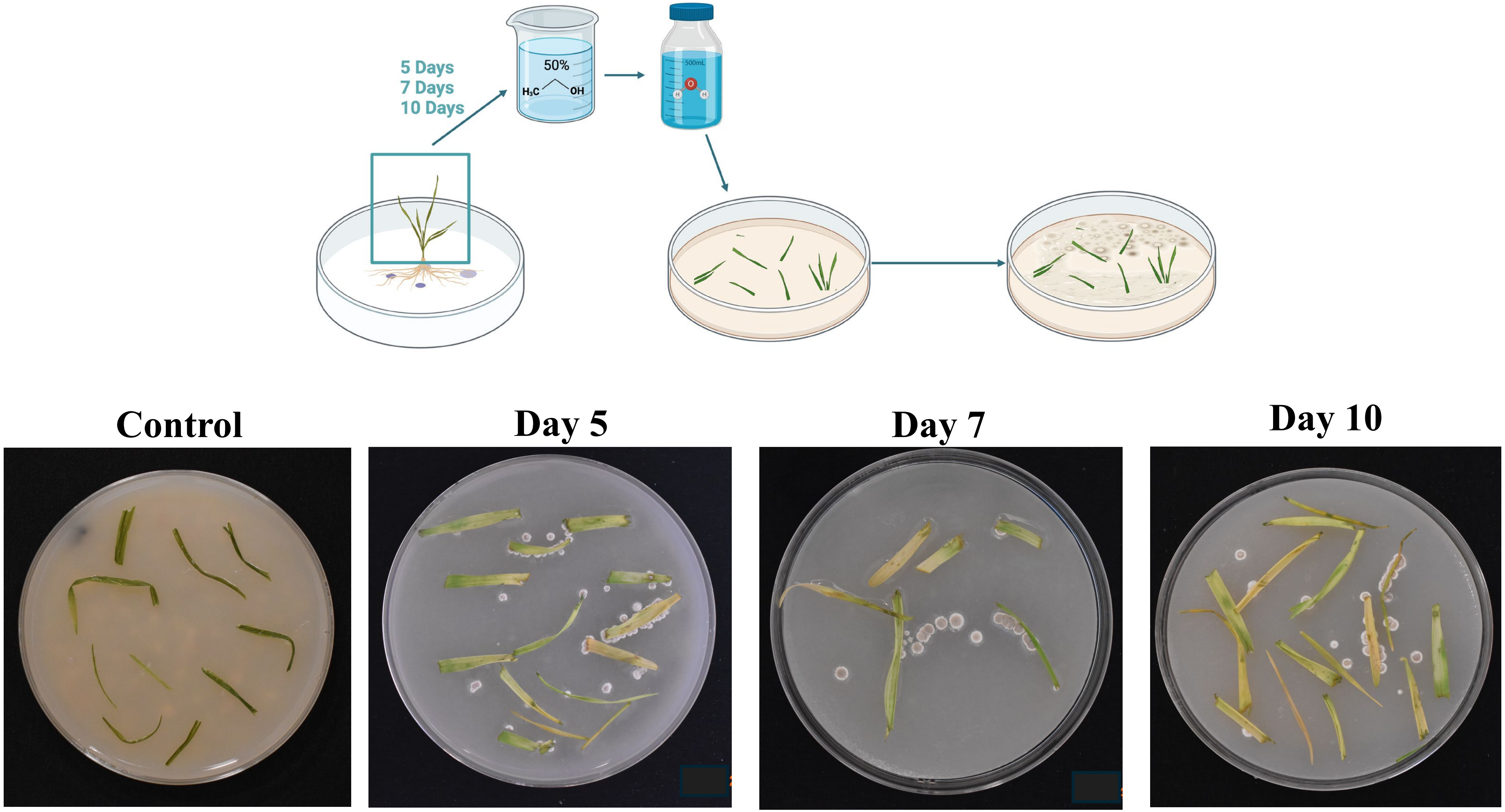

SOM Figure 3 : Protocol developed to evaluate the endophytic lifestyle of *S. coelicolor* in wheat plants. Wheat plants post root inoculation with *S. coelicolor* wee harvested on day 5, 7 and 10, leaf blades from the harvested plants were surface sterilized using 50% ethanol for 15 seconds and were plated on a rich media for *S. coelicolor* colony formations. The control untreated sample on the left depicts plants not treated with *S. coelicolor* and detached leaves were plated on day 10 post culture.
